# “We think this way as a society!”: Community-level science literacy among ultra-Orthodox Jews

**DOI:** 10.1177/09636625221110106

**Published:** 2022-08-01

**Authors:** Lea Taragin-Zeller, Yael Rozenblum, Ayelet Baram-Tsabari

**Affiliations:** The Hebrew University of Jerusalem, Israel; Technion—Israel Institute of Technology, Israel

**Keywords:** community-level science literacy, expertise, Judaism, science and religion, scientific authority

## Abstract

Despite growing interest in community-level science literacy, most studies focus on communities of interest who come together through particular science, environmental or health-related goals. We examine a pre-existing community—ultra-Orthodox Jews in Israel—with a particular history and politics vis-à-vis science, technology, and medicine. First, we show how Haredi cosmologies and culture come together to critique science as an epistemology while engaging with science as a technology. Then, we demonstrate how community-based medical experts serve as both science-related knowledge mediators and gatekeepers. Whereas Haredi Jews are constantly critiqued for their low levels of individual secular and science education, these community-based webs of knowledge seemingly position Haredi individuals with knowledge that surpasses the average “secular” Israeli. This case study develops unique analytical tools in the growing field of community-level science literacy, while pushing forward conversations about self-ascribed experts, knowledge gatekeeping, and the socio-political contexts of group critiques of science.

## 1. Introduction

Despite the wide range of definitions for science literacy, most focus on individual knowledge, attitudes, and competencies (DeBoer, 2000; Laugksch, 2000; Norris and Philips, 2003). These individualistic measures for science literacy have been inspired by the notion that living in the 21st century requires a certain amount of scientific knowledge and understandings on the part of each individual—not only those in the science and technology workforce. Yet, as Lee and Roth (2002) argue:

Fervent defenders of classical notions of “scientific literacy” seem to forget that many children and students are surrounded by adults who not only make a decent living without knowing any science but also proudly proclaim their scientific ignorance. Individuals do well without knowing science because, as an integral part of social life, they have access to different levels of expertise whenever they need it. (p. 34)

These critiques have spurred an interest in community-level science literacy, pushing for an analysis that goes beyond the focus on individual measures of science literacy (Feinstein, 2015; National Academies of Sciences, Engineering, and Medicine, 2016; Roth and Lee, 2002, 2004).

This paper advances current debates in community-based science literacy on two fronts. First, we offer a critical take on the ways “communities” have been described, perceived, and analyzed in community-based literacy studies. Second, venturing toward new understandings of community-level science literacy, we highlight the ways existing structures of knowledge gatekeeping and power effect the development of science literacy at the community level. In doing so, this case study pushes forward conversations about self-ascribed experts, knowledge gatekeeping and the socio-political contexts of group critiques of science.

## 2. The “community” turn in science literacy

For over six decades, scholarship on science literacy has focused on developing ways to measure and advance individual science literacy. These studies were guided by the assumption that (1) in an increasingly technology-based society, science literacy is necessary for the well-being of each individual; (2) science literacy must be taught in the classroom; (3) where teachers must find ways to “help students become competent outsiders to science” (Feinstein, 2011: 168) by equipping their students with tools that will allow them to navigate a technology-based world well beyond the classroom walls (e.g. Basu and Barton, 2007; Bennett et al., 2007).

Science literacy has been measured and assessed in many ways, in educational and non-educational settings, including standardized questionnaires (science indicator GCC) and international tests (e.g. Pisa), as well as using contextualized performance tests (See: Sadler and Zeidler, 2009). Yet, even though science literacy and its assessments has been on the global agenda for decades, the aim to make science accessible to all has brought about little change (Baram-Tsabari, 2022; Rodriguez, 1998; Roth and Lee, 2002). Recent scholarship painfully shows that science education continues to reify existing social inequalities (Canfield et al., 2020; Dawson, 2019). It also shows that scholars have begun to turn away from information deficit models, searching for other cultural and epistemological frameworks to understand the ways people make decisions (Jamieson et al., 2017).

Shifting from an individual framework to community-level science literacy has brought forward fresh ways to analyze the ways people encounter science as part of social and cultural groups, while interpreting and responding to information in ways that reflect communal attitudes and values. To give one example, Kahan (2012) found that attitudes toward climate change tell us less about scientific reasoning and knowledge than about “latent cultural affiliations” (p. 255). Similarly, a Pew Research Center survey found that while more and more Americans are putting climate change as a national priority, Democrats are much more concerned than their Republican counterparts (Kennedy and Johnson, 2020). Not only do people encounter science as members of particular groups, they seek out science through pre-existing networks, utilizing prior acquaintances to help them understand new information.

This resonates with established qualitative research from sociology, anthropology, and history which have demonstrated how engagement with science is repeatedly shaped by social identity, particular historic contexts and power relations (Epstein, 1998; Franklin, 1997; Haraway, 1988; Latour, 1987). As Feinstein and Waddington (2020) put it, “People encounter scientific questions in social context—both *as* members of their social and cultural groups and *with* other members of those groups (p. 156 italics in original).” Not only is the attempt to “filter out” these cultural frameworks unrealistic, they also assume that these frameworks are detrimental to “proper” modes of rational decision-making, while, in reality they can be powerful and constructive resources (Gonzalez et al., 2005). Feinstein and Waddington (2020) suggest that we are all embedded in communities that can (potentially) provide knowledge and expertise:

what stands in the way of the public sphere is not the starkly limited capacity of individuals but the failure of communities to recognize and use their collective strength. (pp. 155–156)

The idea that people encounter science *as* members and *with* members of social and cultural groups is critical to take on board as scholars develop a new set of tools to examine community engagement with science (See also Poltorak et al., 2005). As Snow and Dibner define it, community-level science literacy

does not require each individual to attain a particular threshold of knowledge, skills, and abilities; rather, it is a matter of a community having sufficient shared resources that are distributed and organized in such a way that the varying abilities of community members work in concert to contribute to the community’s overall well-being. (National Academies of Sciences, Engineering, and Medicine, 2016: 6)

Not overlooking the well-established field of the assessment of individual science literacy, scholars of community-level science literacy are currently searching for suitable ways to assess community-level science literacy. As scholars search for new methodological tools, which is not based on aggregated individual scores, this moment is an opportunity for a renewed engagement in other disciplines, such as anthropology, sociology, and science and technology studies.

And still, one of the major challenges for the study of community-based literacy is to offer clear definitions of “community,” as well as more clarity regarding what it means to actually have “sufficient shared resources.” While most scholars have focused on communities of interest or affinity (National Academies of Sciences, Engineering, and Medicine, 2016), which are defined as communities that come together through particular science or health-related goals (such as AIDS and climate change), we analyze community-level science literacy in a setting of a pre-existing religious community.^1^ To develop a more nuanced definition of “community,” we draw on interviews with Haredi (ultra-Orthodox) Jews in Israel to understand how a particular social, cultural, and religious group forms selective uses and understandings of science and technology, together, as a community. While there is no such thing as a homogeneous and unified Jewish Orthodox community in Israel^2^ (nor elsewhere), Haredi Jews are helpful to “think with,” to paraphrase Claude Levi-Strauss (1963), as they do not come together to advance particular science and health-related goals beyond their situated interests.

Continuing recent efforts to go beyond a dichotomous science-religion “conflict” trend, recent scholarship shows that science-related decision-making is negotiated within and through many actors and systems of local knowledge, since both scientific knowledge and socio-religious frameworks serve as “cultural and epistemological tunnels” of science and medicine interpretations, attitudes, and behavior (Taragin-Zeller et al., 2020; Canfield et al., 2020; Goldberg et al., 2019).

And still, most of these studies continuously focus on individual interpretations of religious texts, cultures, and cosmologies. Shifting from an individual framework to a community-based web of science literacy, we focus on the ways people encounter science as part of social and cultural groups, while interpreting and responding to information in ways that reflect communal attitudes and values. In this sense, we are not only interested in how science and medicine are embraced selectively on an individual level but how these decisions are embedded in particular communal networks of science literacy.

Assisted reproductive technologies, for example, have been passionately embraced by religious men and women through creative processes of “koshering medicine” (Ivry, 2010), a rabbinic stamp, which can be essential in making a particular reproductive technology or medical intervention “kosher”^3^ (See also Kahn, 2000). Another recent example shows how amid debates about brain-death definitions, a forum of on-call rabbis called *Arevim* (trustees), emerged in hospitals to have real-time access to both clinical and instrumental reports and thus mediate the medical category of brain-death (Gross et al., 2019: 195).^4^

Building on these instances, we examine how this selective use of technology is grounded in a web of community-based experts, knowledge evaluation and distribution. In this paper, we show how Haredi *askanim*, often translated as “a lay ‘helper’ or ‘doer’” (Kasstan, 2019: 117), have limited science education themselves but nevertheless serve a dual social function—they create a liminal space where “secular” science and medicine is mediated for a religious crowd while also perpetuating insular models of knowledge gatekeeping. These *askanim* are often “a first port of call for Jewish constituents needing advice on affairs relating to healthcare or when lobbying for particular courses of treatment”^5^ (Kasstan, 2019: 117). Following Quast (2018) who states that: “Expert’ is a role that some are selected to play on the basis of all sorts of criteria, epistemic and otherwise” (p. 19), we refer to Haredi *askanim* as self-ascribed experts, as they lack institutionally relevant training and academic or professional competence in medicine. However, they describe themselves and are referred to by Haredi men and women as experts.

Having said that, our focus on webs of community-based systems of knowledge encompasses intra-communal understandings of science, technology, and medicine.^6^ Furthermore, the focus on community-level science literacy reveals how trust in scientific authority is developed as part of overarching state-minority tensions over technological governance (Taragin-Zeller, 2019; Morgan and Roberts, 2012). This is especially crucial for understanding rickety trust in science and scientists (See Bolger and Ecklund, 2018; Jones et al., 2020) among religious groups for whom medical interventions are also situated in a deep state of mistrust in many state-based institutions (Corpuz, 2021).

To be clear, we are not suggesting that all members of religious groups are homogeneous in their attitudes toward science and technology. On the contrary, we suggest looking at how networks of information about science, technology, and medicine are constructed at the group level. We highlight how medical advisors (*askanim*) actively cultivate community-level science literacy among members of a religious minority group with low levels of science education. Whereas Haredi Jews in Israel are constantly critiqued for their low levels of individual secular and science education, according to the self-proclaimed understandings of Haredi medical advisors, community-based webs of knowledge position Haredi individuals with knowledge that surpasses the average Israeli. Not only is this understanding in opposition to public critiques of low levels of science education among Haredim, there is no current system in place to test the scientific knowledge these quasi-medical advisors offer as most have never attended any system of formal science education.

Building on emerging scholarship in the field of community-level science literacy, we push forward conversations about self-ascribed experts, knowledge gatekeeping, and the socio-political contexts of group critiques of science. Examining Haredi Jews in Israel serves as a unique case study to analyze the particular ways groups construct community-level group literacy based on their pre-existing shared identities and goals. We ask: How do Haredi Jews form intra-communal networks of science literacy? How are webs of community-based experts created and sustained? What types of scientific knowledge do they distribute? And, what can these networks of community-based literacy tell us about how scientific authority is formed (and critiqued) in particular group settings?

## 3. Haredim and science: A short history

Haredim (ultra-Orthodox Jews) account for roughly 12% of Israel’s population (Israel Central Bureau of Statistics (ICBS), 2016; Stadler, 2009). Haredi men and women live their lives in accordance with the teachings of the Hebrew Bible (*Tanakh*) which has been reinterpreted through a voluminous body of rabbinic literature, commentary, and Jewish law rulings (*halacha*). Often referred to as an enclave community with strict social and cultural boundaries, Haredim have their own press which is “censored” by rabbinic representatives who tailor daily news to comply with religious values, such as modesty standards which prohibit the public display of female images.^7^

Haredi Jews can be distinguished from Progressive, Conservative, and Religious-Zionist Jewish streams by their self-protective stance and avoidance of secular education and professional training. Beyond their homogeneous appearance, the Haredi sector consists of multiple groups, each with its own religious leaders (rabbis), teachings, and rites (Friedman, 1987; Heilman, 1999; Stadler, 2009; Stadler and Taragin-Zeller, 2017). These can be loosely divided into Lithuanian *yeshiva*-based (Torah learning) communities, Hasidic dynasties, and Sephardi Haredim (who trace their origins to the Iberian peninsula, North Africa and the Middle East).

Scholars have emphasized the importance of Haredi formal education, leading to what Friedman (1987) termed “a society of learners.” Haredi society views Torah study as the main trajectory to piety for men, while marginalizing other activities, including employment and secular studies (Stadler, 2009; Stadler and Taragin-Zeller, 2017). Since the establishment of the Israeli state, Haredim in Israel have acquired varying levels of autonomy from national curriculums and regulation systems (Perry-Hazan, 2015). Alongside Israel’s national education system, an independent Haredi school system was developed to bypass subjects that pose challenges to intra-communal worldviews and lifestyles.^8^

Today, Haredi education is made up of segregated K-12 schooling for boys and girls, followed by an extensive network of institutions of higher religious study for men (*yeshivot*). Haredi schools aim to prepare children for their gendered roles in society—to assist boys to become religious scholars, and to prepare girls to support their husband’s Torah study as breadwinners and domestic caregivers. Hence, STEM (science, technology, engineering, and mathematics) subjects are sparsely taught in ultra-Orthodox schools. As Haredi women are expected to become wives, mothers, and main breadwinners, they typically study math and science up to the age of 15. Boys, on the contrary, rarely study science or math beyond fifth or sixth grade (ages 11–12), as this is perceived as unnecessary knowledge for a life of “pure” Torah scholarship (Manny-Ikan and Rosen, 2013).

In the past, there have been numerous efforts to introduce basic scientific learning into the curricula by the Israeli government. Even though these were mostly repelled by political pressure, in recent years, minimal core studies have been gradually introduced through a number of school reforms (Katzir and Perry-Hazan, 2019). Many Haredi groups, and especially their leadership, oppose national testing which makes it almost impossible to conduct comparative assessments. We do know that in the 2018 PISA tests, Haredi girls had lower scores compared to their secular counterparts in Israel,^9^ mirroring Haredi-secular knowledge gaps which persist in higher education as well.

As Haredim are projected to make up 25% of the Israeli population by 2065 (ICBS, 2016), integration and engagement in secular education and professional studies are heavily linked with anxiety among policy-makers regarding the future face of the Israeli state. Even though Haredi Jews engage with science-related information in their everyday life and make decisions based on these encounters, to date, little work has thoroughly examined perceptions of science and technology beyond the walls of classroom education (Franken and Levrau, 2020). These anxieties informed state initiatives before COVID-19 began, but their importance was made even more clear in 2020/1. During the pandemic, Haredi Jews were slower to adhere to social distancing guidelines than other groups in Israeli society (Waitzberg et al., 2020).

By the end of March 2020, the epidemiological disaster was clear: there were significant clusters of infections in Haredi neighborhoods, 40%–60% of all coronavirus patients at four major hospitals, even though they make only 12% of Israel’s population. Haredi Jews were also slow to vaccinate. Among the first wave of vaccinations which were offered to men and women above the age of 60, 49% of Haredi men and women vaccinated compared to the national average of 64% (Halbfinger, 2020; Krombi and Berenblum, 2021). Even though public concern regarding Haredi attitudes toward vaccination were part of heated debates in New York, Brussels, and London, especially in the wake of the 2018–2019 measles outbreak, little work has paid attention to the underlying networks of scientific and health decision-making among Haredi populations (Kasstan, 2021; Taragin-Zeller et al., 2020).

## 4. Methods

This research is part of a large mixed-methods study devoted to understanding the ways Haredi Jews consume and negotiate scientific information, as individuals and as a group. To be clear, we do not intend to measure or assess the level of science literacy among Haredi Jews. Our intention is to analyze how perceptions of community-level science literacy shapes the ways Haredi Jews engage with bio-medicine in a context where each individual has limited science education. The data include surveys and in-depth interviews, yet the findings presented in this article largely draw on qualitative data collected between 2020 and 2021 in Israel. Twenty in-depth interviews were conducted with 10 Haredi men and 10 Haredi women, who affiliated with one out of the three main Haredi sub-groups Sephardi, Lithuanian, and Hasidic Haredim. Participants were recruited through a snowball method. Five men worked in some capacity as medical *askanim*, which allowed us to capture the ways these community experts create and distribute scientific knowledge. All participants attended Haredi schooling and their education reflected the gendered Haredi norm: seven women attended secular higher education and worked outside of their homes in a variety of white-collar positions, whereas most men did not have any higher secular education and combined Torah learning with low-income positions, primarily as teachers and third-sector charity workers.

COVID-19 impacted the research methods we employed. Before COVID-19, eight interviews were conducted face-to-face. After providing background information, each interview was asked about the ways they consume science and make health-related decisions. As we continued to collect data between November to February 2021, we tailored the research methods to COVID-19 social distancing guidelines. As most Haredim do not use Zoom or other online video platforms, phone interviews were utilized. Following this procedure, an additional 12 interviews were conducted.

Interviews were recorded (when participants permitted), transcribed verbatim, and analyzed on both a separate and comparative basis. Thematic analysis was used in order to analyze the findings. This method is intended to develop understandings about what is common among a set of data, “from the bottom up” (Taylor and Bogdan, 1984). The first step entails classifying the data into different themes and then combining and cataloging related patterns into sub-themes. At this point, individual experiences are pieced together to form a comprehensive picture of a collective experience.

To ensure reliability, three co-authors individually examined the data and refined the themes that were generated from thematic analysis. While this article focuses on science and religion and science and everyday life, other themes included: state-religion tensions regarding required secular curricula, attitudes toward Internet use, science news consumption, science and religion, literacy and skills, science-related decision-making, source evaluation, science experiences in childhood, religious authority, and science in everyday life. All names in this article are pseudonyms, other than Rabbi Firer, who is a public figure and the data which appear in this article about him are already part of the public sphere. Ethical approval was obtained from the ethics committee at the Technion Institute of Technology.

## 5. Findings

### Science as an epistemology, science as a technology

Haredi Jews should not be conflated with other religious groups, like the Amish, who disengage from “Western” medical treatment. On the contrary, to solidify their use of medicine and technology, there are many interpretations of Jewish law that perceive the use of medical care as a religious obligation. “For your own sake, therefore, be most careful” (Deuteronomy 4:15), the requirement of self-preservation—requires Jews to guard their health while engaging in all that “Western” bio-medicine can offer. For example, Rivka, a women in her mid-thirties and a receptionist in a medical clinic in a Haredi neighborhood in Jerusalem shared how:

When I was young, I dreamed I would become a doctor one day. Today, I know that that dream will never happen. My only way into the medical system would have to be through the back door.

Rivka reveals how doctors and medicine are perceived as unachievable but desired professions. And, still, we found a paradoxical situation that constantly came up in our data: on one hand, many of the participants held science, and especially its use in the context of “Western” bio-medicine in high regards. On the other hand, there is no overarching acceptance of scientific authority over science-related issues. Yossi, a 34-year-old school teacher, offered a similar stance. He said:

The Haredi society . . . puts into people’s roots that science is wisdom, but the true and big wisdom is Judaism, and science is part of small wisdoms.

According to Yossi, then, there is no conflict between science and religion because the hierarchy is crystal clear. Science is characterized as a “small wisdom.” While this categorization signifies a partial acceptance of scientific knowledge, this knowledge is secondary to the “true” wisdom of Judaism. In other words, the wisdom of the Torah is the litmus test for other types of wisdom. This perception of science, as Yossi put it, is “put into people’s roots,” a metaphor which captures how deep and entrenched these perceptions really are. As ideas about science are constantly constructed as “small wisdom,” there is an element of science appreciation, on one hand, but a constant hesitancy, on the other hand. To make sense of this intellectual dissonance, we argue that Haredi cosmologies and culture come together to critique science as an epistemology while engaging with science as a technology. As Rivka, a Sephardi 36-year-old educator with three children who resided in Bnei Brak explained:

I don’t think we need to be scared of it. We can use it in a smart and right way. I don’t worry about it. They are just tools, that is technology.

According to Rivka, technology can be used, albeit in a “smart and right” way. Rivka goes on to explain how, for example, with proper rabbinic supervision, even in vitro fertilization (IVF) babies can be allowed in Judaism (See: Ivry, 2010; Kahn, 2000). But as an epistemology, science cannot be fully relied on. It is crucial to say that this distinction between science as a technology and science as an epistemology is a fictive binary distinction. In real life, there are no clear boundaries between science-based applications and the epistemologies that birthed them into being. Yet, the “use” of this distinction allows for a clear and cut division of hierarchy between “wisdoms.” According to this conceptualization, if Yossi would refer to science as a “big wisdom,” it would threaten the supreme wisdom of the Torah. Hence, constructing science as a “small wisdom,” as a technology, removes this potential threat. In some cases, science and religion can work hand in hand, and in others the threat of science must surrender to the “true wisdom.” Many of the participants offered critiques of science and primarily of scientists as a reason for their rickety trust in science, resonating with Ecklund and Bolger’s findings (Bolger and Ecklund, 2018). Shlomi, for example, explained that:

Each researcher or scientist sits and thinks about something, but that doesn’t mean that it is really like that. So what? That is what *they* say. What are you going to do about that? Scientific news is just rumors. (italics in original)

Shlomi’s narrative reflects a clear-cut boundary-making between “them” and “us.” Furthermore, scientific “news” are not held on a pedestal according to Shlomi. This critical understanding of science reflects either a knowledge lacuna about the accepted modes of scientific research, or a deep mistrust. As Ayala Fader (2021) put it: “Ultra-Orthodox Jews are not necessarily anti-science. Rather, some Ultra-Orthodox Jews are suspicious about certain social and political channels where scientific knowledge is produced and legitimated.” Other interlocuters also critiqued scientific motivations per se. For example, Sruli, a full-time Yeshiva student said:

Science is a type of wisdom with many mistakes . . . sometimes there are also people who are not just and they publicize their studies for recognition and for money. There are many people who think this way, but we think this way as a society.

Many of our respondents, like Sruli, linked their personal views to their society or as Yossi put it earlier, their “roots.” But, what does it mean to “think this way as a society”? And, how is this type of “thinking” cultivated? In the next section, we describe how these “roots” or ways of thinking are circulated and enabled through a network of Haredi experts who form the basis of a community-based science literacy web of knowledge. While most Haredi Jews have little background in science, these networks allow Haredi Jews to engage with science in their everyday lives while feeling like they have access to all necessary knowledge.

### Community networks of science literacy

All Israeli residents are entitled to basic health care as a fundamental right. In addition to medical services offered by the Israeli ministry of health, there has been a growth of charity-based Haredi organizations that offer consultation and support in medical arenas. To bolster their authority, many organizations are founded by public figures who cultivate both rabbinic and medical expertise or, alternatively, have rabbis on their boards to supplement rabbinic authority to their growing medical expertise. As many Haredim believe that the true wisdom resides in the Torah (“Big wisdom,” as we explained in the first section), a rabbinic stamp can be essential in making a particular technology or medical intervention “kosher.”

Organizations such as “*Yad Sarah*” (The Hand of Sarah), “*Ezra Lemarpheh*” (Assisting the sick), and “*Magen Lechole*” (Protecting the sick), typically operate in tandem with Israel’s state-provided medical system, while offering tailored support to Haredi men and women. While Rabbi Firer’s^10^ organization “*Ezrah Lemarpeh*” and the other medical charities do not limit their clientele to Haredi men and women, the narrative they put forward is one which highlights the ways they can address the particular challenges and sensitives Haredi men and women navigate as part of their encounters with Israel’s “secular” medical system. As Rabbi Friedman, the founder of one such organization explained during an interview: “We offer our services to everyone. Because we are Haredi, we tend to attract Haredim. We also offer particular services that Haredim need. Like, help on the Sabbath. But we are really here for everyone!”

Over the years, these organizations have cultivated a network of Haredi medical experts, often referred to as medical *askanim.* In Hebrew, the word *asakim* means business, and *askanim* refers to anyone who handles business-related matters. There are different types of *askanim*, which in our case includes Haredi medical experts (typically men) who serve as intra-communal authorities, offering support and advice to mitigate Israel’s medical system. For example, “*Ezra Lamarpeh*” is a non-profit medical support organization founded by Rabbi Firer in 1979. Their website states that:

It handles thousands of emergency calls and has become Israel’s leading medical referral expert. Rabbi Firer’s up to date knowledge in many areas of medicine has led him to develop a data bank on the world’s top medical specialists. People come to the Rabbi, not only for help in medical diagnosis, but for advice where to best be treated and by which doctor.^11^

Rabbi Firer, perhaps one of the most well-known and highly regarded Haredi medical consultants (as he defines it), has no medical training but has devoted his life to finding ways to help people receive the best treatment possible. While some critique has been voiced by Israeli doctors, he was awarded a national prize for his contribution to society by the state of Israel. And yet, when he was offered to light a torch in Israel’s official Independence Day ceremony, he declined. This refusal ignited age-old tensions regarding Haredi citizenship in Israel, primarily tensions around Haredi ambivalence around Zionism, the limited levels of secular education in Haredi schools and their exemption from compulsory military service. Rabbi Firer’s decline symbolized that while his efforts to better the individual lives of Israeli citizens are well-received, the incorporation of Haredi Jews in Israel’s public life remains unresolved.

As mentioned earlier, there have been heated debates among Israeli policy-makers regarding the incorporation of STEM studies within Haredi education. As Haredi men and women have very little science education, these medical organizations occupy a liminal space, where Haredi Jews (predominantly men) develop ideas and expertise about science, technology, and medicine, but these organizations also signify another growing area of Haredi-state tension—the field of healthcare.

While policy-makers advocate for more science education as a basic need for all, these organizations offer a communal solution to the low levels of individual science education, as Rabbi Stasky, who works in one of these organizations described it:

At a certain age—Haredim understand medicine more than secular Jews, and I beg for forgiveness for over-generalizing. [Why do you think so?] I don’t think! I am stating a fact. The reason is that in the Haredi sector we take medicine very seriously because of the value of life . . . But, on the other hand, on an individual level—especially young couples—they have a very low level of knowledge, I would even say too little, really no knowledge. I met a woman who came out of a surgery of a womb amputation but thought it was just a vaginal resection. But on a general level—whoever has a medical problem—because of the abundance of medical organizations, the *askanim* of our society help people understand. They make the relevant medical information accessible.

Rabbi Stasky offers a creative way to solve the problem of limited Haredi science education. While Israeli policy-makers advocate for more STEM education to benefit individual Haredi men and women, according to Rabbi Stasky this is not necessary as these organizations offer a communal solution. While this may be perceived as a defensive claim to protect the alternative system maintained by *askanim*, utilizing this web of experts offers the necessary knowledge, which, according to him, surpasses the knowledge of secular Jews. Even though he is well aware of the gaps in individual knowledge (demonstrated by his critique of the low levels of individual medical literacy), Rabbi Stasky is not troubled by these knowledge gaps as members of the Haredi community have access to communal levels of science literacy. Drawing on recent developments in science education, we suggest viewing these intra-communal knowledge networks as a form of community-level science literacy. Indeed, our data show that during moments of need Haredi men and women contact these organizations and heed to their advice when necessary. As Chaim Lev, a 44-year-old medical *askan* from Petah Tikvah explained:

People come back from the doctor and they can’t understand anything. I specialize in helping people with genetic-related challenges—and you know, the secretaries there don’t have time. There is a black hole and people need help from someone outside the clinic. With genetics, there is a lot of talk about percentages and this needs to be translated, Because genetics is not exact science, something the doctors exaggerate and then we need to explain to them what to worry about and what not. It is a daily challenge.

Chaim, who has no formal science education, is one of the *askanim* who help couples read their medical documents and refer them to the best help possible. Not only does he translate the medical texts, he also helps people make sense of these results “explaining what to worry about and what not” while dismissing the doctors for “exaggerating” and critiquing the statistical infrastructure of genetics for not being “exact science.” To be clear, in no way do we critique Chaim’s intentions. He, and many other people we spoke to, is really trying to assist people who are struggling with severe health issues. However, their perceptions of “necessary” knowledge seem to be at odds with medical professionals.

This system also creates a culture of second opinions. As Rabbi Stasky put it: “Even though we do not have individual high-levels of education, because we value life we always ask for a second opinion! And, because all cancer patients call Rabbi Firer, the medical knowledge accumulates.” Hence, while this model allows knowledge to “accumulate” within the community, this constant mediation created a culture of hesitancy regarding medical knowledge as it highlights conflicting intentions and “exaggerated” truth claims. As Efrat, a Haredi woman, who works in an Israeli hospital shared:

At the hospital, there is a Haredi woman who has severe COVID-19 and is ventilated in the intensive care unit. Sarah, is a Hasidic woman, from one of the Hasidic courts in vicinity to the hospital. A member of her community heard from one of the *askanim* of a substance that dilutes fat, which supposedly can help COVID-19. He shared this information with the *Admor*, the rabbinic leader of the community who said they should give this medicine to Sarah. Following his suggestion, Sarah’s eight children have been calling the hospital all day, requesting that their mother receive this medicine. I don’t know what to tell them. There is no way the doctors will give medicine that has not been approved. But they claim that it works.

While this particular event is specifically connected to COVID-19, it brings to the fore many of the familiar tensions between religion and science, medical authority and religious authority, and individual versus communal power (Fader, 2020; Gross et al., 2019; Taragin-Zeller, 2019, 2021). These types of tensions have been documented by ethnographers of science and medicine, both in the context of medical care (Ivry and Teman, 2019; Kasstan, 2019; Raucher, 2020) and in the context of legal-ethical disputes, such as organ donation and brain-death (Gross et al., 2019). Yet, in the context of community-based literacy, it also raises important questions about self-ascribed expertise and the ramifications of community-based networks that come into conflict with institutionalized medical systems.

While seeds of these critiques were visible pre-COVID-19, especially during the last wave of vaccine hesitancy (Kasstan, 2022a, 2022b), COVID-19 brought these alternative cultures of Haredi medicine and health organizations to the forefront of public debate as Haredi Jews were slower to adhere to social distancing guidelines and slower to vaccinate amid the pandemic, reflecting “how public health emergencies reveal multiple ideas of immunity between health services and religiously Orthodox minorities” (Kasstan, 2022a: 4). There were also some groups, particularly Hasidic Haredi groups in Israel, the United Kingdom, and the United States, who decided to pursue herd immunity to avoid having to close religious institutions, in direct opposition to state guidelines.^12^ The contagious element of the coronavirus, thus, demonstrates how an examination of alternative systems of health and medicine is crucial to the entire society and that understanding how community-based literacy evolves and operates is critical to us all, now more than ever.

## 6. Discussion

Despite the breadth of definitions for science literacy, they typically focus on a particular measure that captures individual knowledge, attitudes, and competencies (DeBoer, 2000; Laugksch, 2000; Norris and Philips, 2003). In this article, we draw on recent calls to go beyond the focus on individual knowledge by highlighting community-level systems of science literacy in a particular religious setting. Drawing on the case study of Haredi Jews, we show how science and medical literacy are developed at a community level. Science literacy raises different implications for the areas of bio-medicine raised in this article, such as genetics, COVID-19, and public health. Future studies could attend to drawing out the intersections and divergences between different areas of bio-medicine (cf. Kasstan, 2022a).

In what follows, we attend to a striking paradox that is evident from our data. Whereas Haredi Jews in Israel have extremely low levels of individual secular and science education, according to their own understandings, community-based webs of knowledge position Haredi individuals with knowledge that surpasses the average Israeli due to their community-level literacy. Having said that this understanding relies on the subjective reports people shared, an issue we further develop below.

### What counts as sufficient community-science literacy?

This case study raises a few key interventions that can help enhance and develop community-science literacy, both as a methodological tool and as a field of study. First, one of the major challenges for the study of community-based literacy is to offer clear definitions of what we mean when we talk about “community.” While many have focused on communities that come together through particular science or health-related goals, we examined a pre-existing community with a particular socio-political stance *vis-à-vis* science, technology, and medicine. Thus, even though Haredim constitute many sub-groups, most would self-identify as Haredi, which makes the task of delineating group boundaries for the purposes of study, rather simple. But, it also raises important comparative questions for future studies, such as: do different types of communities construct community-level science literacy in different ways? Does the fact that some groups have pre-existing shared identities and goals affect the ways science-literacy is imagined and achieved?

This study also brings to the forefront questions regarding the quality and level of science literacy each individual can access, and what this knowledge means for them. For example, if we go back to the story about the woman who had no idea what type of surgery she actually went through, this division of knowledge raises serious questions about the types of information individuals have access to and how these may affect the ways they navigate the health system as well as their own processes of understanding and decision-making. In other words, we must create tools to determine what it means to actually have “sufficient” shared resources.

Another questions that arises from our analysis is—how should we analyze the place self-ascribed community-based experts play in the cultivation of community-level literacy? What type of measures do we have in place to estimate their backgrounds and alternative models of expertise? We know that struggles over science and science authority are deeply entrenched in structures of power (Benyei et al., 2021). How do we navigate these when attempting to define levels of scientific expertise for experts who gained their expertise beyond the halls of conventional “Western” science education, but are, nevertheless, attempting to interpret this knowledge for members of their communities?

One of the clear findings of our study is that many of our respondents used an uncritical tone toward *askanim*. This finding was further supported by Rivka Neriya-Ben Shahar et al. (2022) who conducted an extensive study of attitudes toward the Israeli health system among Israeli Haredim. Based on 11 focus groups with 126 Haredi men and women, they found many stories of rabbinic “saviors,” as they put it (e.g. the doctor was wrong, but the rabbi told me what to do and he was right!) compared to almost no stories which criticized rabbinic guidance in issues of medicine. The dominant discourse that arises from their findings is a constant appraisal of rabbinic knowledge who often rely on *askanim* to navigate the “secular” health systems. In this article, we are trying to understand how these knowledge systems operate.

Furthermore, in the case at hand, almost all of these experts are male, which is surprising due to the lower levels of science education among Haredi men. Hence, while these organizations offer relevant knowledge and expertise, these networks also perpetuate insular models of gendered knowledge gatekeeping (see: Taragin-Zeller, 2021). Our analysis showcases how medical *askanim* cultivate their scientific knowledge and expertise outside of institutionalized science education programs. While scholarship has shown how Haredi men volunteer in health-based organizations as alternative ways to perform male citizenship in lieu of army participation (Stadler, 2009), this study shows that “secular” knowledge about science and medicine is achieved through their work in health-related charity organizations.

Having said that, scholarship on community-level science literacy must find fresh ways to think about pre-existing histories and politics that particular communities have regarding science. As a first step, scholarship could likely draw on the vast sociological and anthropological scholarship that pays attention to structures of power within the politics of science (Gorur et al., 2019; Hilgartner et al., 2015). However, this would need to be developed and situated in particular community-state dynamics, which can vary from place to place and from different scientific topic to another.

Finally, shifting the focus of analysis from individual science literacy to community-level science literacy, also allows us to pay attention to science-religion relationality in fresh ways. While much research on science and religion has tended to focus on legal and theological objections to science (Gross et al., 2019; Inhorn, 2003; Ivry, 2010), our article shows how religious groups, and especially religious and ethnic minority groups engage in science in selective and critical ways. Vaccinated patterns amid COVID-19 have been a clear flag to these selective and critical group attitudes, highlighting the urgency of this topic (see Kasstan, 2022a).

These attitudes are not merely a product of pre-existing codified laws but are constantly nurtured by a network of medical *askanim* who collectively create a community-based set of knowledge and understanding about science. As people make health and science-related decisions, *askanim* mediate “secular” knowledge which is treated with suspicion, until it is deemed either “kosher” or rejected by these quasi-authoritative men. Thus, this analysis highlights the importance of studying science mediators among religious and ethnic minority groups as hubs of knowledge that go beyond the sum of their parts. As we have shown, their community-level science literacy greatly differs from the aggregate collection of individuals. On the contrary, in this case, community-level science literacy reifies existing structures of knowledge gatekeeping and power. If future studies succeed in understanding how diverse communities engage with science, perhaps we will be able to better understand the mechanism by which some scientific claims are accepted (or rejected) by particular groups.

## Notes

1. Notwithstanding the political differences between communities of interest whose coming together is also linked to ideas of equity and social justice (especially in the context of HIV), we bring these case studies together to ask questions about what “community” means in the context of community-level science literacy.

2. Even though there is a Jewish rabbinate commonly recognized as the supreme rabbinic authority in Israel, in practice the boundaries of these communities are constantly debated. Nevertheless, Haredi Jews typically perceive of themselves as a particular group, compared to other streams of Judaism.

3. Making reproductive technology “kosher” is linked to rabbinic maneuvers around biomedical technologies to regulate assistive reproductive technologies, such as IVF, in order to fit with religious laws (especially procreation).

4. For other examples of social science research exploring religious authorities and bio-medicine, see Kahn (2000), Raucher (2020), Fader (2021), and Neriya-Ben Shahar (2022).

5. For more on Askanim see Kasstan (2019).

6. While these concepts are analytically distinct, as we show in this article, in real life, these topics are negotiated in tandem.

7. Cohen (2012), Golan and Mishol-Shauli (2018), and Neriya-Ben Shahar (2016).

8. See Taragin-Zeller and Kasstan (2022a) for an analysis of sex education among Haredim in Israel and the United Kingdom.

9. Unfortunately, we do not have the same data for Haredi boys. Due to small numbers of Haredi boys who took this test, their scores do not appear in the final PISA results. https://cms.education.gov.il/educationcms/units/rama/school_evaluation/dochotmaarachtim.htm

10. In contrast to all other names in this paper which have been changed to protect participant identities, we use Rabbi Firer’s his real name as he is a well-known public figure.

11.
https://www.ezra-lemarpe.org/en

12.
https://www.ynetnews.com/magazine/article/H1pCKTpAv
